# Tacrolimus Toxicity due to Biliary Obstruction in a Combined Kidney and Liver Transplant Recipient

**DOI:** 10.1155/2017/9096435

**Published:** 2017-01-09

**Authors:** Samuel Chan, Michael T. Burke, David W. Johnson, Ross S. Francis, David W. Mudge

**Affiliations:** ^1^Queensland Renal Transplant Service, Princess Alexandra Hospital, Brisbane, QLD, Australia; ^2^School of Medicine, The University of Queensland, Brisbane, QLD, Australia

## Abstract

The immunosuppressant tacrolimus has a narrow therapeutic window, necessitating therapeutic drug monitoring to maintain efficacy and minimise toxicity. There are very few reports examining the impact of impaired biliary excretion on tacrolimus blood levels or toxicity. We report the case of a 26-year-old combined liver and kidney transplant recipient, who developed acute biliary obstruction leading to tacrolimus toxicity with very high blood tacrolimus levels. Despite a careful evaluation, no alternative cause was found for her acute kidney injury, and her kidney function returned to previous baseline within several days following treatment of the biliary obstruction and temporary withdrawal of tacrolimus.

## 1. Introduction

Tacrolimus is a calcineurin inhibitor immunosuppressant with a narrow therapeutic window [1, 2], necessitating therapeutic drug monitoring to maintain efficacy and minimise toxicity [3, 4]. It is lipophilic and insoluble in water and is a substrate for the drug efflux pump p-glycoprotein and the metabolising enzymes cytochrome P450 3A4 and cytochrome P450 3A5 [3, 4]. Metabolites are excreted primarily in bile [1]. We report the case of a recipient of a combined liver and kidney transplant with stable allograft function on tacrolimus-based immunosuppression, who developed biliary obstruction due to retained gallstones after a cholecystectomy. She rapidly developed signs of tacrolimus toxicity, predominantly central nervous system toxicity, in association with a sudden increase in her blood tacrolimus level. We postulate that the biliary obstruction led to the acute elevation of tacrolimus levels and toxicity, having excluded other possible causes.

## 2. Case Report

A 26-year-old female liver and kidney transplant recipient presented with a forty-eight-hour history of fever and right upper quadrant pain. She denied any recent illnesses, medication changes, or other symptoms. Her medications included tacrolimus 2.5 mg twice daily, mycophenolate mofetil 360 mg twice daily, prednisolone 7.5 mg daily, trimethoprim/sulfamethoxazole 80/400 mg daily, cholecalciferol 25 *μ*g daily, ranitidine 150 mg twice daily, and magnesium 500 mg twice daily. Her past medical history included end-stage liver disease due to biliary atresia, which was treated with liver transplantation three years prior to presentation. She had undergone cholecystectomy two years prior to the current admission for cholelithiasis complicated by acute cholangitis. She had no other prior surgeries. Her postoperative course was uncomplicated and she made a complete recovery. She subsequently developed end-stage kidney disease due to biopsy-proven diffuse proliferative glomerulonephritis and had received kidney transplantation thirteen months prior to presentation. Her transplant was a five-human-leukocyte antigen mismatch. There were no donor specific antibodies or any antibodies present on Luminex screening. The initial therapy was standard immunosuppression at that time (tacrolimus, mycophenolate mofetil, and prednisolone). The kidney transplant course had been complicated by early delayed graft function secondary to acute tubular necrosis and suspected antibody-mediated rejection, with presence of peritubular capillaritis but absent C4d, which was treated successfully with a six-week course of plasma exchange. Her subsequent serum creatinine concentrations had remained stable at approximately 1.5 mg/dL.

On examination, she was febrile at 39.5°C, hypotensive with systolic blood pressure of 98 mmHg, and tachycardic at 120 beats per minute in sinus rhythm. She maintained normal oxygen saturation on room air. Her abdomen was tender in the right upper quadrant, with no palpable organomegaly or rebound tenderness. Bowel sounds were audible. There was no abnormality found on examination of her cardiovascular or respiratory systems.

**Figure 1:**
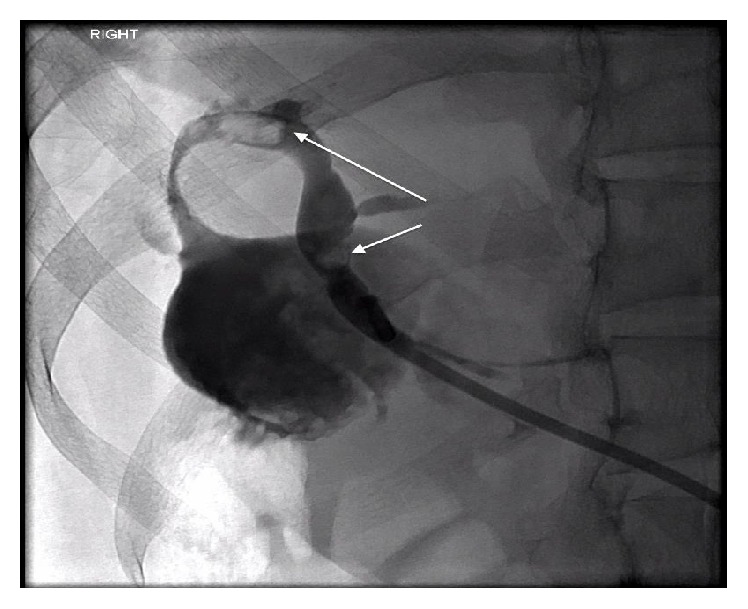
Cholangiogram demonstrating filling defects representative of biliary stones (arrows) present in the common bile duct.

Laboratory investigation demonstrated worsening graft function with a serum potassium concentration of 5.7 mmol/L, serum creatinine concentration of 2.2 mg/dL, and a trough blood tacrolimus level of 5.6 ng/mL. Her serum total bilirubin was 6.4 mg/dL, serum alkaline phosphatase was 187 U/L, gamma-glutamyl transferase was 370 U/L, alanine transaminase was 242 U/L, and aspartate transaminase was 190 U/L. Her C-reactive protein was 158 mg/L. BK virus DNA was not detected in her serum. Blood and urine cultures were sterile. Computed tomography of her abdomen and pelvis showed dilatation of the intrahepatic biliary tree within the lobar liver transplant raising the suspicion of possible biliary-enteric anastomotic stricture. Magnetic resonance cholangiopancreatography and cholangiogram revealed biliary obstruction, with intrahepatic biliary duct dilatation, due to the presence of biliary stones in the main bile duct and near the biliary-enteric anastomosis (Figure 1). The presentation was consistent with cholangitis and, hence, the patient was initially treated with fluid resuscitation with normal saline and intravenous piperacillin/tazobactam.

**Figure 2:**
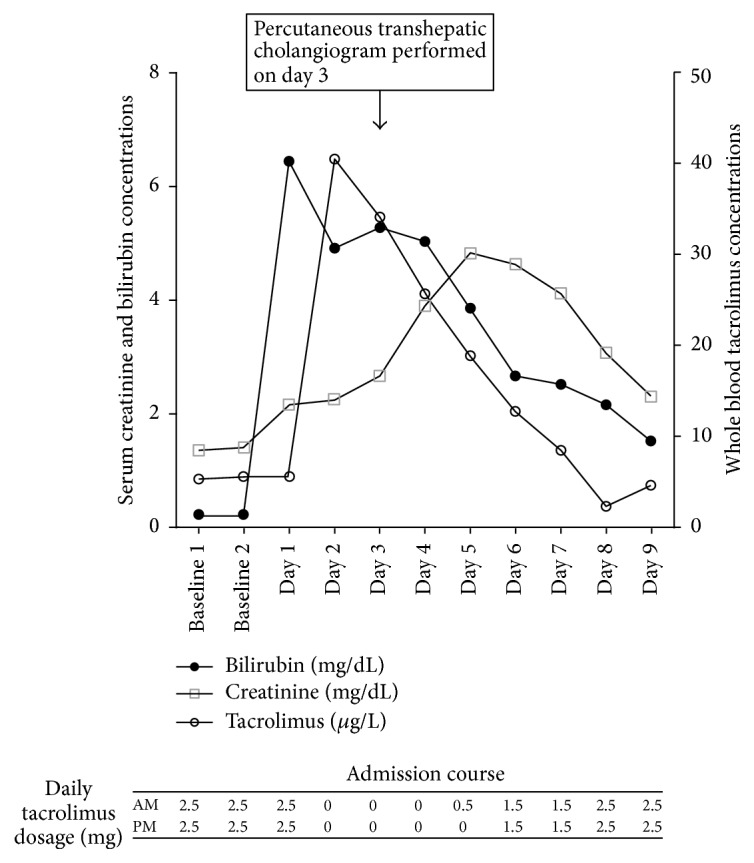
Serum bilirubin, creatinine, and tacrolimus concentrations and total daily tacrolimus dosage throughout patient admission.

On admission day 2, her serum creatinine and trough blood tacrolimus concentrations rose markedly to 2.77 mg/dL and 45 ng/mL, respectively, in the absence of any new medications apart from her antibiotics (Figure 2). The immunoassay used to measure tacrolimus levels in this patient was High Performance Liquid Chromatography with tandem mass spectrometric detection. This technique is not susceptible to the interference from tacrolimus metabolites [5]. Her serum total bilirubin was 108 mg/dL, serum alkaline phosphatase was 985 U/L, gamma-glutamyl transferase was 785 U/L, alanine transaminase was 654 U/L, and aspartate transaminase was 585 U/L. She concomitantly developed nausea, vomiting, tremors, blurred vision, hypertension, and delirium without any focal neurological signs. Her tacrolimus was withheld for seven doses and then recommenced at a dose 0.5 mg twice daily (80% reduction of original dose). Tacrolimus levels progressively fell and reached a target level of 8.7 ng/mL by day 7. Her symptoms resolved by day 5. Biliary drainage was restored by day 3 via percutaneous transhepatic cholangiography.

## 3. Discussion

The patient described in this case report developed clinical features suggestive of acute tacrolimus toxicity, manifested by neurotoxicity, gastrointestinal upset, hypertension, and acute kidney injury, in the setting of acute biliary obstruction. The close temporal association between the rise and fall of the patient's serum tacrolimus levels and the onset and relief of her biliary obstruction strongly suggested that biliary obstruction per se led to changes in serum tacrolimus concentrations, particularly as there were no concomitant interfering medications, medication dosing errors, or preceding gastrointestinal upset. Furthermore, the associations between the rise and fall of her serum tacrolimus concentrations and the respective onsets and offsets of her nausea, vomiting, tremors, blurred vision, hypertension, delirium, and acute kidney injury suggested that these clinical manifestations represented tacrolimus toxicity. Whilst intravenous piperacillin/tazobactam may potentially interact with tacrolimus, there is minimal supporting evidence in the literature regarding this drug-drug interaction; hence, antibiotic mediated tacrolimus toxicity is an unlikely mechanism in this case.

Tacrolimus is metabolised extensively in the liver by the cytochrome P450 family enzymes (particularly CYP3A4 and CYP3A5) with approximately 95% of tacrolimus metabolites being excreted in the bile and only 2% excreted via the urine [6]. In contrast to cyclosporin, the bioavailability of tacrolimus is not appreciably reduced by the absence of bile in the intestine [7]. Consequently, biliary obstruction and/or hepatocyte dysfunction would be hypothesised to impair hepatic metabolism and biliary excretion of tacrolimus but not intestinal absorption. Whilst it has been suggested that biliary obstruction may lead to elevated serum tacrolimus levels [8], reports of this in the literature are scarce. In an experimental dog model, bile duct ligation was shown to greatly increase the area under the plasma concentration versus time curve following orally administered tacrolimus, but not following intravenously administered tacrolimus [7]. Furthermore, bile duct ligation was found to effectively increase the bioavailability of oral tacrolimus 3- to 4-fold, possibly by impaired presystemic metabolism of tacrolimus [7]. Moreover, in a single-centre, retrospective observational study of 35 liver transplant recipients, Kobuchi et al. [9] reported that postoperative biliary stricture was associated with marked intra- and interpatient variability in the tacrolimus trough level normalised dose, which was positively correlated with serum bilirubin level. These findings support the contention that biliary obstruction may substantially increase blood tacrolimus levels, as evidenced by this foregoing case report.

In conclusion, this case report illustrates the potential for serious elevation of blood tacrolimus levels and clinical toxicity in the presence of biliary obstruction. The diagnosis of biliary obstruction should therefore prompt clinicians to closely monitor tacrolimus blood levels and look for clinical signs of toxicity on a daily basis, with appropriate reduction of dosage when necessary. Similarly, following relief of biliary obstruction, frequent monitoring is required for several days thereafter and tacrolimus dosage may need to be increased. All clinicians (transplant and primary care) should be aware of this important clinical interaction.
